# Biology and ecology of the brown dog tick, *Rhipicephalus sanguineus*

**DOI:** 10.1186/1756-3305-3-26

**Published:** 2010-04-08

**Authors:** Filipe Dantas-Torres

**Affiliations:** 1 Dipartimento di Sanità Pubblica e Zootecnia, Facoltà di Medicina Veterinaria Università degli Studi di Bari, 70010 Valenzano, Bari, Italy

## Abstract

The brown dog tick (*Rhipicephalus sanguineus*) is the most widespread tick in the world and a well-recognized vector of many pathogens affecting dogs and occasionally humans. This tick can be found on dogs living in both urban and rural areas, being highly adapted to live within human dwellings and being active throughout the year not only in tropical and subtropical regions, but also in some temperate areas. Depending on factors such as climate and host availability, *Rh. sanguineus *can complete up to four generations per year. Recent studies have demonstrated that ticks exposed to high temperatures attach and feed on humans and rabbits more rapidly. This observation suggests that the risk of human parasitism by *Rh. sanguineus *could increase in areas experiencing warmer and/or longer summers, consequently increasing the risk of transmission of zoonotic agents (e.g., *Rickettsia conorii *and *Rickettsia rickettsii*). In the present article, some aspects of the biology and ecology of *Rh. sanguineus *ticks are discussed including the possible impact of current climate changes on populations of this tick around the world.

## Review

Ticks (suborder Ixodida) are the most important group of vectors of pathogens within the phylum Arthropoda, being comparable only to mosquitoes (family Culicidae) [1,2]. They are responsible for the maintenance and transmission of many pathogens affecting domestic animals and humans, including several species of bacteria, helminths, protozoa, and viruses [3].

**Figure 1:**
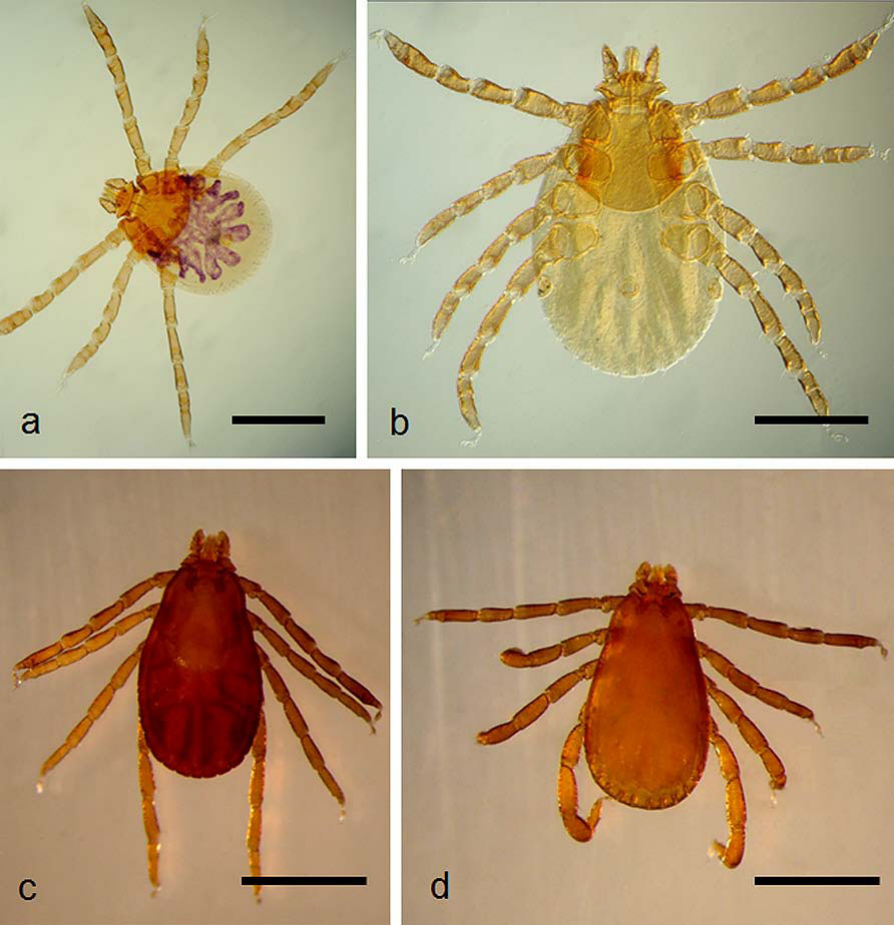
**Immature and adult stages of *Rhipicephalus sanguineus***. A: larva (mounted in Hoyer's medium; bar = 400 μm). B: nymph (mounted in Hoyer's medium; bar = 0.5 mm). C: female (bar = 1 mm). D: male (bar = 1 mm).

The brown dog tick *Rhipicephalus sanguineus *(Figure 1) is the most widespread tick in the world, even considering that many ticks currently identified as *Rh. sanguineus *might actually represent other closely related species (e.g., *Rhipicephalus turanicus*). This tick is a parasite of dogs that can occasionally parasitize other hosts, including humans. Moreover, *Rh. sanguineus *is a vector of many disease agents, some of them (e.g., *Coxiella burnetii, Ehrlichia canis*, *Rickettsia conorii*, and *Rickettsia rickettsii*) being of zoonotic concern [4].

Due to its veterinary and public health relevance, *Rh. sanguineus *is one of the most studied ticks. Indeed, a number of studies on its biology and ecology have been carried out in many parts of the world. Certainly, knowledge of the natural history of this tick is seminal for a better understanding of the eco-epidemiology of tick-borne diseases, such as Mediterranean spotted fever and Rocky Mountain spotted fever. Herein, some aspects of the biology and ecology of *Rh. sanguineus *are discussed, including the possible impact of current climate changes on populations of this tick around the world.

## Biology of *Rhipicephalus sanguineus*

### Ethology

From an ethological standpoint, *Rh. sanguineus *is an endophilic (adapted to indoor living), monotropic (all developmental stages feed on the same host species), and three-host (each life stage requires a new host to feed on) tick species. However, although highly endophilic, *Rh. sanguineus *is also able to survive in outdoor environments, mainly if refuges (e.g., limestone walls) are available. Moreover, although monotropic, this tick can occasionally feed on other hosts (e.g., humans), which do not belong to its 'natural trophic chain'. These facts indicate that *Rh. sanguineus *is a catholic tick, being able to adopt different strategies for survival, as needed.

When seeking a host, the brown dog tick is a hunter (host-seeking behaviour), although it can also adopt the ambush strategy (questing behaviour). Indeed, all these behavioural patterns exhibited by *Rh. sanguineus *have been acquired throughout its evolutionary history. Perhaps, these traits of this tick have evolved from its relationship with the domestic dog and their shared environment, being part the tick's strategy for survival and perpetuation.

### Attachment, feeding and mating

Once on the dog, *Rh. sanguineus *uses its chelicerae to pierce the host's skin and then inserts its hypostome and chelicerae into the host's epidermis, occasionally reaching the upper layers of dermis [5]. During attachment, the tick secretes a cement-like substance, which forms a cone on the surface of epidermis that extends up to the *stratum corneum *[5]. While probing for blood, capillary and small blood vessels are lacerated and haemorrhage occurs, creating a feeding pool [6], from which the tick sucks blood and other fluids (telmophagy).

The feeding period of *Rh. sanguineus *can vary from two days (e.g., larvae) to several weeks (e.g., females), depending on tick developmental stage (e.g., feeding period of nymphs is longer than that of larvae) and host (e.g., engorgement of females may take longer on rabbits than on dogs) [7,8]. Male ticks can take multiple blood meals. Indeed, it has been shown that male ticks previously attached to one dog can move onto another co-housed dog and feed on it [9]. Furthermore, male ticks can remain for long periods of time on the host. Interestingly, it has been observed that the presence of males can increase the feeding performance of *Rh. sanguineus *immature ticks, particularly nymphs [10]. This fact suggests that males may have other biological roles in addition to reproduction.

**Figure 2:**
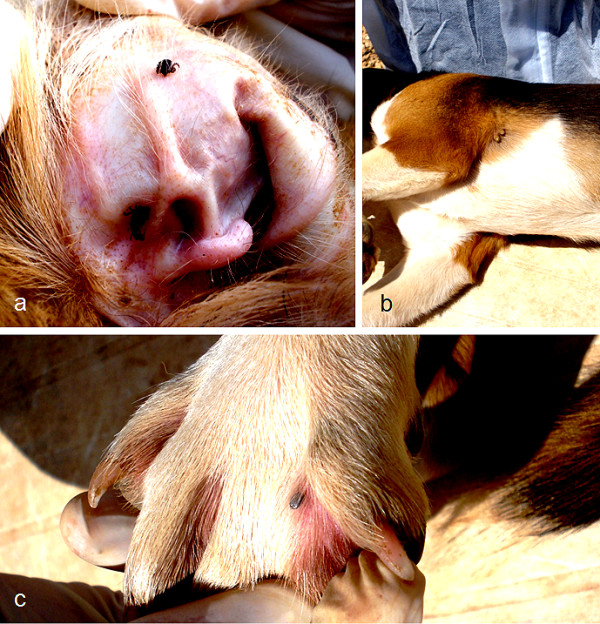
**Attachment sites of *Rhipicephalus sanguineus***. A: three adults on the ear of a dog. B: two females attached to the axilla of a dog. C: an engorged nymph on the interdigital region of a dog.

**Figure 3:**
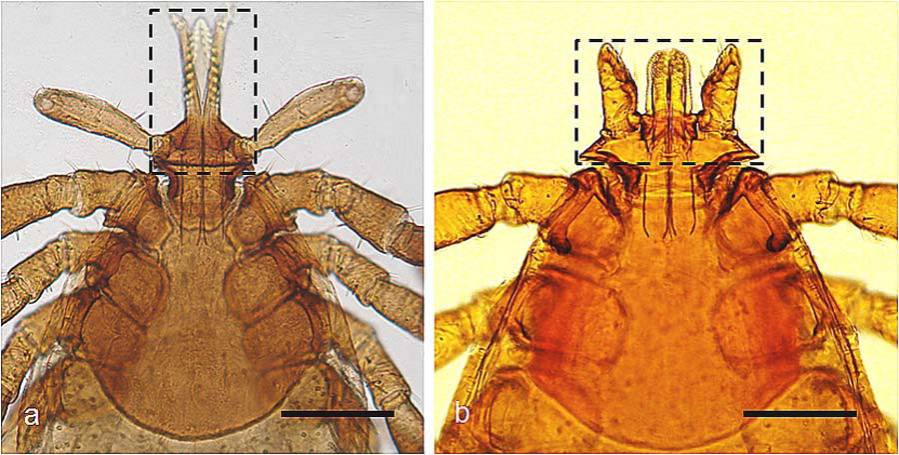
**Tick mouthparts**. A: *Ixodes ricinus *nymph (bar = 200 μm). B: *Rhipicephalus sanguineus *nymph (bar = 250 μm). Note the *rostrum *of *Rh. sanguineus *(wider than long) in comparison with the one of *I. ricinus *(twice longer than wide).

**Figure 4:**
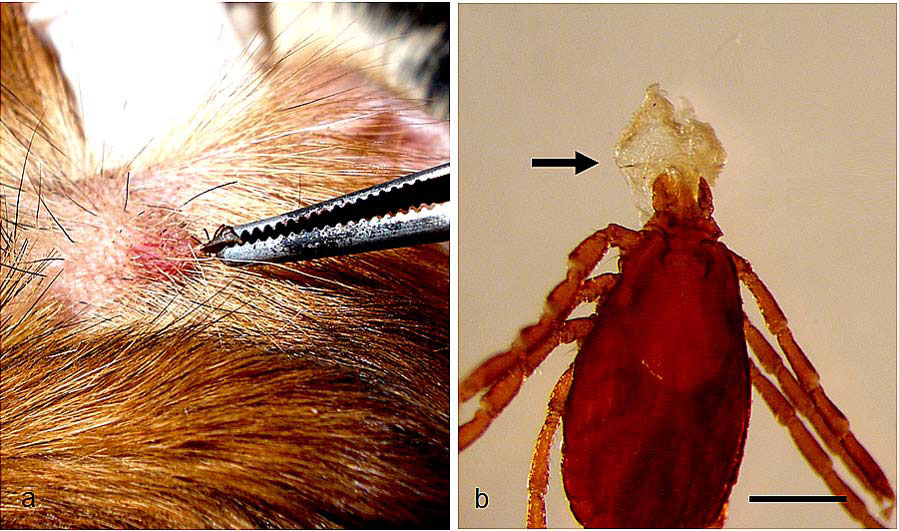
**Attachment of *Rhipicephalus sanguineus***. A: A male firmly attached to the dog's skin. Note that while the tick is being gently pulled with the help of a tweezers, the skin is stretched out. B: A female exhibiting a piece of a dog's skin that remained attached to her mouthparts after her forced removal.

*Rhipicephalus sanguineus *ticks can attach everywhere on the dog, but the head (particularly on ears), inter-digital spaces, back, inguinal region, and axilla (Figure 2) are among their preferred attachment sites [11-16]. Although *Rhipicephalus *ticks have short hypostome (Figure 3) and attach more superficially in comparison with others ticks (e.g., most species of *Amblyomma *and *Ixodes*), they can attach firmly to the host's skin (Figure 4).

**Figure 5:**
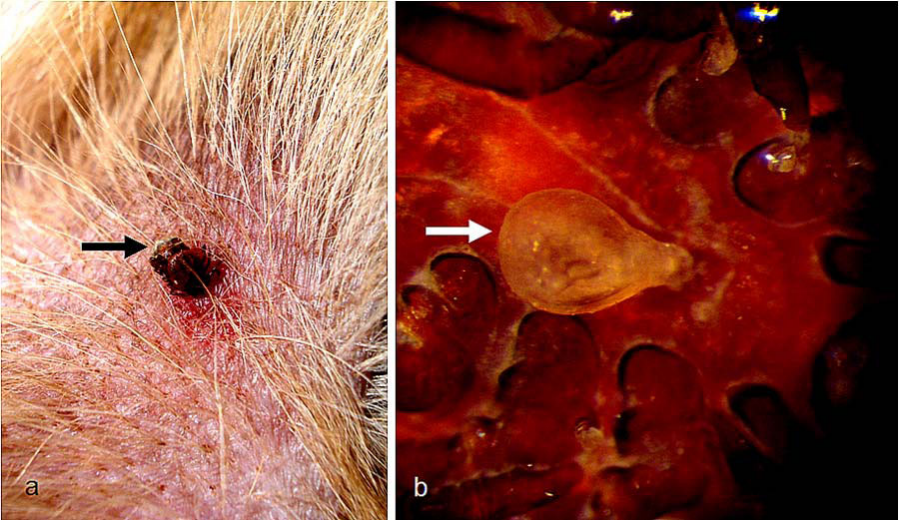
**Mating of *Rhipicephalus sanguineus***. A: A couple of *Rh. sanguineus *mating on a dog (the male is arrowed). B: A spermatophore attached to the female genital aperture (bar = 600 μm).

As a metastriate tick (lineage Metastriata), *Rh. sanguineus *attains sexual maturity and mates solely on the host. Although the female can start to feed even in the absence of a male, she will not become fully engorged unless mated. Indeed, the ingestion of blood is a major stimulus for spermatogenesis in males and for oogenesis in females. During mating, the male climbs onto the dorsum of the female and crawls to her ventral surface, standing in juxtaposition (venter to venter) with her. Then, the male stimulates the female genital aperture (gonopore), by inserting the tips of his chelicerae into it. Soon afterwards, the male transfers the spermatophore (a double-walled, sperm-filled sac) to the female genital aperture (Figure 5) with the help of his mouthparts [17]. The spermatophore then everts itself into the female's genital tract. Around 24 h after copulation, a capsule full of mature spermatozoa (spermiophores) can be found in the *receptaculum seminis *of dissected females [17].

### Drop-off rhythm

Most ticks have a definite circadian rhythm of detachment from the host (drop-off), which is usually coordinated with host's activity [18]. *Rhipicephalus sanguineus *larvae exhibit a diurnal drop-off pattern [19-21], detaching mostly during the daytime. Conversely, engorged nymphs and females detach predominantly during the night period [19-21]. The reasons for this particular drop-off behaviour of larvae, nymphs, and females of *Rh. sanguineus *are not fully understood, but might be related to the activities of the host as well as it might represent strategies adopted by the tick during different phases of its life cycle. In any case, this data should be taken into account while planning control measures focused on the environment, as the places where dogs stay at night are more likely to harbour the largest number of non-parasitic stages of *Rh. sanguineus *[21].

### Female oviposition and egg hatching

**Figure 6:**
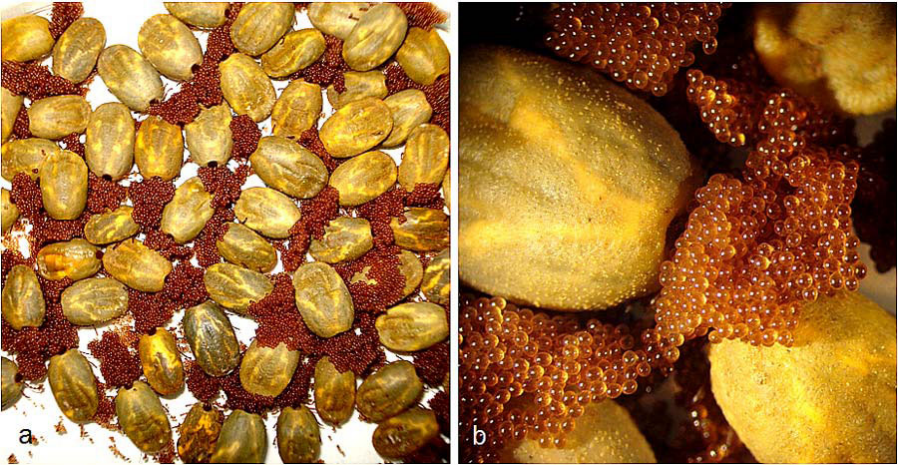
**Oviposition of *Rhipicephalus sanguineus***. A: Several females laying eggs under laboratory conditions (temperature 26°C, relative humidity, 80%). B: A close-up of the previous image, showing in detail the newly laid eggs.

When feeding is complete, the engorged female detaches from the host, drops to the ground and after a pre-oviposition period (from three days to some weeks) deposits thousands of eggs (Figure 6). Typically, females of *Rh. sanguineus *oviposit uninterruptedly an average of 1500-4000 eggs [7,22]; however, some disturbed females (e.g., removed daily from the vials for separation and counting of the eggs) can interrupt the oviposition and then restart it the day after, although loses in terms of egg production efficiency are usually minor (unpublished observations). The oviposition period can last for several weeks and the number of eggs laid by each female is directly correlated with her weight and the length of the oviposition period [7]. Eggs are deposited in hidden places, such as cracks and crevices in the walls, between rocks, and sometimes, almost inside the ground. The females need to find a hidden place to protect themselves and their fore coming progeny, as they constitute an easy prey for predators, such as spiders [23], birds [24], and wasps [25].

The egg hatching is preceded by an incubation period that ranges from 6 days to some weeks [4]. Similarly to what occurs in other tick species, a longitudinal fissure (hatching line) encircling the egg chorion can be observed at the end of the incubation period, characterizing the beginning of the hatching process, which culminates in the hatching of a flat, fragile six-legged larva. The newly hatched larva usually needs sometime to harden its chitin-made exoskeleton before seeking a host. For instance, in an experimental study, larvae younger than 7 days were unable to attach and feed on rats [22].

### Moulting process

When feeding is complete, engorged larvae and nymphs detach from the host and drop to the ground to find a hidden place. The moulting process is preceded by a period of seclusion (pre-moult period) that might vary widely (from days to several weeks), depending on factors such as life stage (i.e., it takes longer in nymphs than in larvae) and weather conditions (e.g., stressful temperature and humidity can extend the moulting period). At low temperatures (e.g., at 10°C), the engorged larvae and nymphs may undergo diapause and the higher is the temperature, the shorter is the moulting period [26].

**Figure 7:**
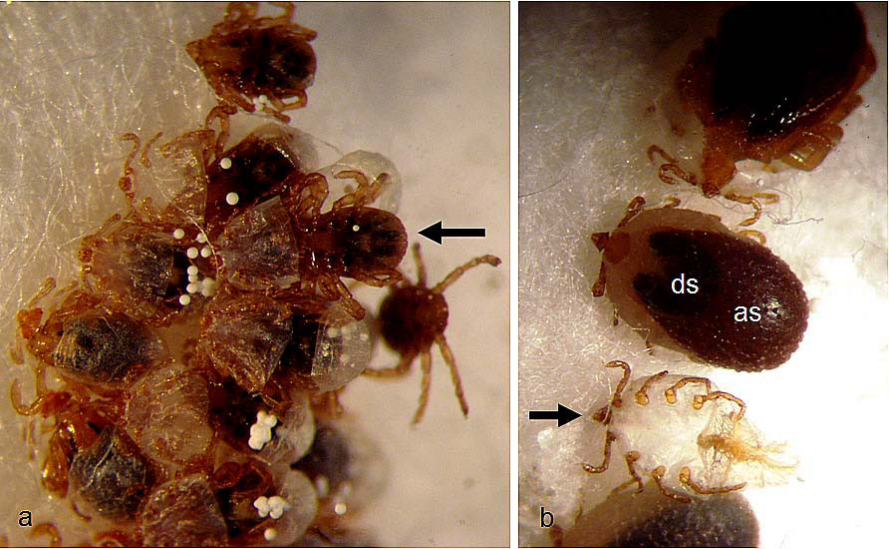
**Moulting of *Rhipicephalus sanguineus***. A: A nymph (arrow) emerging from its larval exuvia. B: An engorged nymph (few hours prior the ecydisis), exhibiting the short, anterior dorsal *scutum *(ds) and the *alloscutum *(as) of a typical female. A nymphal exuvia (arrow) left behind by other female can be seen as well.

As in insects, the ecydisis in ticks is regulated by moulting hormones (ecdysteroids) [27]. In *Rh. sanguineus*, the ecydisis starts with the rupture of the old cuticula and then the old integument is moved forward by means of abdominal peristaltic waves (see additional file 1). In a few hours, the newly moulted tick emerges, leaving behind its exuvia (Figure 7).

During moulting, even prior to rupture of and emergence from its old integument, the tick starts to defecate. The faeces are initially seen as white spherules (see additional file 2) consisting of guanine, xanthine and other similar compounds [28]. These compounds result from the metabolism of the blood meal and are formed in the Malpighian tubules as the nitrogenous wastes, being accumulated in the rectal sac and eliminated via the anal pore [28]. Guanine is the most abundant component of tick excreta and is a natural semiochemical that has been identified as an assembly pheromone, inducing aggregation in many *Ixodes *and argasid species [28]. So far, neither guanine nor other assembly pheromones have been identified for *Rh. sanguineus *ticks. What is known is that aggregation accelerates the moulting process of nymphs [29]. Interestingly, the presence of newly moulted nymphs appears to act as a mechanical stimulus for the ecdysis of other nymphs (see additional file 3) (unpublished observations).

## Ecology of *Rhipicephalus sanguineus*

### On-host ecology

The domestic dog is the main host of *Rh. sanguineus *in both urban and rural areas [30-32]. Occasionally, *Rh. sanguineus *can infest a wide range of domestic and wild hosts, including cats, rodents, birds, and humans [33-39]. The parasitism by *Rh. sanguineus *on hosts other than dogs is quite unusual in certain areas, being mainly associated to the presence of heavily infested dogs and in highly infested environments. In the same way, ticks collected from domestic and wild animals that might eventually resemble *Rh. sanguineus *might actually represent other species, such as *Rh. turanicus *which is often found on cattle, horses, goats, cats, and a wide range of wildlife species [36].

The likelihood of a host other than the dog being attacked by *Rh. sanguineus *might vary according to tick population. For instance, the human parasitism by *Rh. sanguineus *is relatively common in Europe, particularly during the summer [40]. In contrast, the human parasitism is much less common (or maybe much less reported) in South American countries [41], such as Brazil [38,42].

The prevalence and mean intensity of infestation by *Rh. sanguineus *on dogs can vary widely, both geographically and seasonally. These and other "on-host" ecological parameters can also vary according to diverse factors, at both population (e.g., dog population density and proportion of dogs treated with ectoparasiticides or tick repellents within a population) and individual levels (e.g., age, breed, and lifestyle). For instance, the prevalence of *Rh. sanguineus *infestation on dogs can be as high as 80% in some areas, as in north-eastern Thailand [43]. The prevalence is higher among free-ranging dogs (which are usually untreated against ectoparasites) as compared with domiciled dogs [31]. Mean intensities of infestation of 3.8, 5.4, 7.8 and 39.4 have been reported in north-western Georgia (United States) [44], north-eastern Brazil [32], south-eastern Brazil [45], and Italy [46], respectively. In south-eastern Brazil, the prevalence and mean intensity were much higher among dogs living in houses with grassy yards as compared with dogs kept in apartments [45]. In a recent study carried out in the same region, dogs were significantly more infested during the dry season [15]. Furthermore, the tick burden is often higher among urban dogs in comparison with rural ones [30,32,47]. However, in some rural areas, *Rh. sanguineus *might be even absent and dogs can be infested by many other tick species (e.g., *Amblyomma oblongoguttatum*, *Amblyomma ovale*, and *Amblyomma cajennense *in eastern Amazon, Brazil) [48].

It is not rare to see some dogs infested by a single tick and others confined in the same kennel (even in the same cage) carrying hundreds of ticks. This suggests that the tick burden might also be influenced by individual dog factors, such as age and breed. Indeed, the tick burden is heavier on young dogs in comparison to older ones [16,32]. Young dogs heavily infested by ticks might develop anaemia, particularly if they are also infected by tick-borne pathogens, such as *Ehrlichia *spp. [49]. Although the prevalence of infestation is often higher among males than females [15], it is uncertain whether this is a gender-related susceptibility or a matter of exposition. Furthermore, some breeds (e.g., English cocker spaniels) are apparently more susceptible than others [50]. A more recent study has suggested that *Rh. sanguineus *ticks can display distinct behavioural patterns upon exposure to odours from different dog breeds [51]. As a hunter tick, *Rh. sanguineus *seeks its host actively oriented by host-produced substances (kairomones), including CO_2_. Whether other host-produced substances can induce questing activity or even an escape-oriented behaviour in *Rh. sanguineus *remains uncertain.

The resistance of dogs to ticks is usually measured by comparing some biological parameters of ticks fed on tick-naïve dogs with those fed on dogs previously infested by ticks [50,52]. These biological parameters (e.g., tick yield, weight of engorged females and egg production efficiency) can provide direct or indirect evidence on the resistance of dogs to ticks. However, even though some females fed on dogs previously exposed to ticks might weigh significantly less and produce fewer eggs than those fed on tick-naïve dogs, these females will still be able to produce viable offspring.

A recent study showed that *Rh. sanguineus *ticks fed on resistant hosts (i.e., guinea pigs) presented several histological alterations (e.g., swelling of the epithelial cells of Malpighian tubules, an increase in guanine content secreted by Malpighian tubules, vacuolization of epithelial wall of tracheae, and vacuolization of oocytes) as compared to ticks fed on dogs [53]. However, further research employing ultrastructural and immunohistochemical techniques would be helpful to reveal the nature of these alterations.

### Off-host ecology

Strange as it seems (e.g., when you see a single dog infested by hundreds of ticks), most of the ticks are not on the dog but in the environment. As a typical three-host tick, *Rh. sanguineus *spends most of its lifetime in the environment, where it is under direct influence of several biotic (e.g., predators) and abiotic (e.g., weather condition) factors.

In tropical and subtropical areas, *Rh. sanguineus *ticks are prevalent throughout the year [42,54,55] whereas in temperate regions they are most active from the late spring to early autumn [56,57]. *Rhipicephalus sanguineus *ticks can overwinter in the environment and even infest dogs during winter in some regions of temperate climate (e.g., south-eastern Oklahoma and north-western Arkansas, United States) [11]. However, successful oviposition, egg hatch as well as larval and nymphal moulting are unlikely at low temperature conditions [26,58]. In this regard, it has been shown that *Rh. sanguineus *can develop well under different conditions in terms of temperature (e.g., 20-35°C) and relative humidity (e.g., 35-95%) [26].

The number of generations that *Rh. sanguineus *ticks can complete each year can vary from region to region. Under favourable conditions (e.g., temperature, relative humidity, and host availability), they can complete up to three or four generations per year, as recorded in centre-western Brazil [14,15].

**Figure 8:**
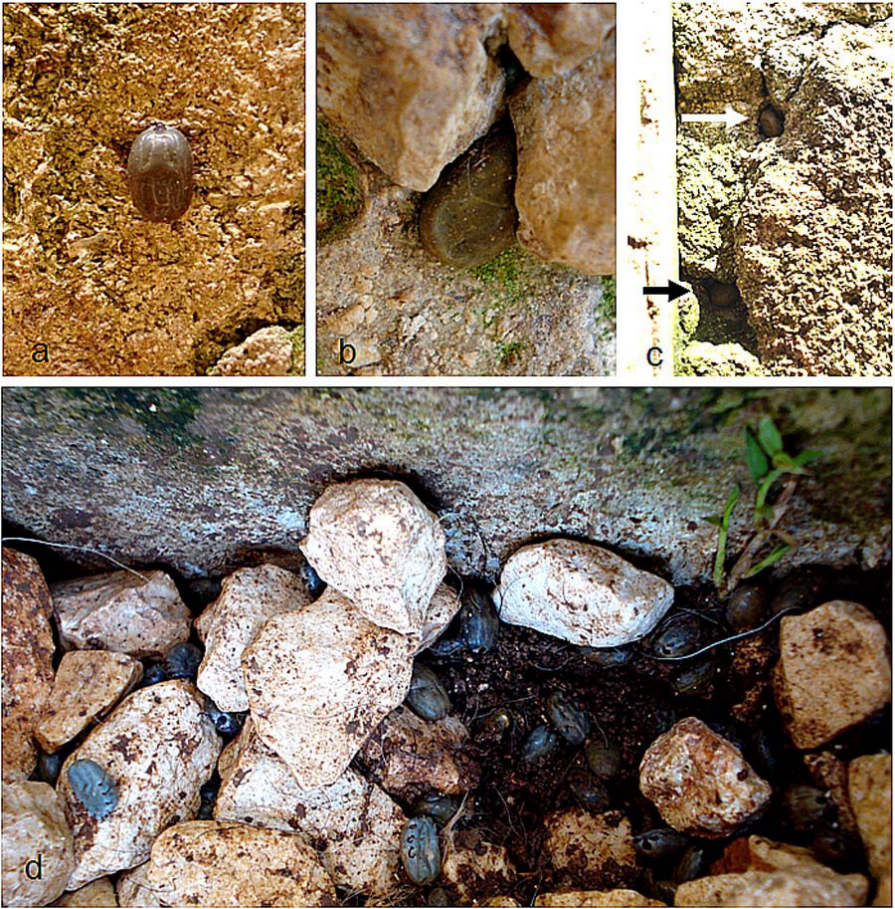
**Hiding-places of *Rhipicephalus sanguineus***. A: A fully engorged female walking on a limestone wall. B and C: Engorged females (arrows) hidden in cracks of the same wall. D: Several engorged females on the ground between rocks.

*Rhipicephalus sanguineus *is an endophilous tick, being usually found indoors crawling on carpets, walls, and furniture [38,59]. However, it can also be abundant in peridomestic areas, as reported in eastern Arizona [60,61]. They can be found walking on outside walls of houses, on the ground (between rocks), and inside cracks and crevices (Figure 8). Indeed, high levels of environmental infestation might increase the risk of human exposure to *Rh. sanguineus *[38,59,62,63] and thus the risk of acquiring certain tick-borne pathogens, such as *R. rickettsii *[59].

In an epidemiological study carried out in Marseille (France) it was observed that dense centres of housing were much less favourable for *Rh. sanguineus *ticks than scattered ones [64]. Furthermore, it was observed that houses with gardens were more a suitable biotope for *Rh. sanguineus *than the environment of large buildings [64]. Similar results have been obtained in Japan, where dogs that had contact with a garden (two weeks prior to examination) had a higher chance of being infested by *Rh. sanguineus *[65]. Furthermore, in the same Japanese study, this tick was most frequently associated with dogs from urban and suburban areas [65].

Overall, studies on the ecology of *Rh. sanguineus *show that this tick is well-adapted to live within human dwellings, being also capable to colonize peridomiciliary environments (e.g., gardens and kennels) if the weather is suitable and if hosts are available.

## The brown dog tick, global warming, human parasitism, and tick-borne diseases

The brown dog tick is an ectoparasite of public health significance, being involved in the transmission of major human pathogens, as it is the case of *R. rickettsii *[66]. There has been a lot of discussion about climate changes and their impact on ticks and on the eco-epidemiology of tick-borne diseases [67]. Tick biology and ecology are under the direct influence of climate factors, such as temperature and humidity. Indeed, while global warming might affect the survival of some tick species that are adapted to live in humid environments (e.g., Atlantic rainforest), it will probably have only a minor (if any) negative impact on ticks like *Rh. sanguineus *that are less dependent upon a moisture-rich habitat for survival [68] and more resistant to desiccating conditions [26]. On the contrary, the global warming might prompt the establishment of tick populations in previously free areas. For instance, it has been speculated that an increase of about 2-3°C in the mean temperature from April to September could result in the establishment of populations of *Rh. sanguineus *in regions of northern temperate Europe [67]. However, the actual impact of global warming on *Rh. sanguineus *ticks is uncertain.

Interestingly, recent studies have demonstrated that *Rh. sanguineus *ticks exposed to high temperatures attach more rapidly to rabbits and humans [40,69]. Therefore, it has been suggested that the risk of human parasitism could increase in areas that are experiencing warmer and/or longer summers, which could ultimately increase the risk of transmission of some pathogens, such as *R. conorii *[40]. It is important to stress that exposure to light and high temperature provoke excitation and induce increased questing behaviour not only in *Rh. sanguineus*, but in any tick species, particularly in those parasitic on homeothermic vertebrates.

Cases of human parasitism by *Rh. sanguineus *ticks have sporadically been described in the literature [38,59,62,70-76] and the risk factors associated to this parasitism include dog ownership, presence of infested dogs indoors and high level of environmental infestation. In Brazil, people dealing daily with dogs (e.g., veterinarians, pet shop workers, and dog owners) appear to be at risk of exposure to *Rh. sanguineus *[38,42]. In south-east Nigeria, in an outbreak of human parasitism by *Rh. sanguineus*, the grounds of the family dwelling, the sheep pens, and dog kennels were heavily infested by *Rh. sanguineus *[63]. Indeed, the higher is the level of environmental infestation, the higher is the risk of human exposure to *Rh. sanguineus *ticks.

## Concluding remarks and research needs

Dogs can be affected by a number of vector-borne diseases [77,78], most of which are transmitted by ticks. Among the tick species implicated in the transmission of pathogens to them [79-81], *Rh. sanguineus *is undoubtedly the most important species from the veterinary standpoint. Moreover, in the era of globalization and climate changes, the brown dog tick has becoming increasingly relevant from a public health perspective. This tick has also been implicated in the transmission of pathogens of zoonotic concern (e.g., *R. rickettsii*) and recent studies have shown that *Rh. sanguineus *ticks exposed to high temperatures are more prone to bite humans [40]. This scenario highlights that the climate warming could affect *Rh. sanguineus *populations of around the world and, consequently, the epidemiology of certain tick-borne infections [40].

Another important issue to be considered is the taxonomy of the genus *Rhipicephalus *and, in particular, of the *Rh. sanguineus *group that has long been a subject of research [82,83]. The main problem is that the type-specimen of *Rh. sanguineus *has been lost [82] and, therefore, a *bona fide *taxonomic definition of this species is currently lacking. This taxonomic question needs to be resolved in the near future to avoid misidentifications and misleading on the role of *Rhipicephalus *spp. ticks in the epidemiology of tick-borne diseases.

As previously mentioned in this article, some dog breeds appear to be more resistant than others [50] to infestations by *Rh. sanguineus*. Further studies on the possible role of individual dog factors (e.g., genetics and nutritional status) on the susceptibility of dogs to ticks are needed. Specifically, it would be interesting to investigate whether previous tick infestations could reduce the number of successive tick bites and thus the risk of infection by tick-borne pathogens, for example, *E. canis *and *Babesia vogeli*.

Although dogs are the main hosts of *Rh. sanguineus*, the finding of this tick on wild canids [37] indicates that free-ranging wild canids might be involved in its maintenance and dispersion through different regions. This could have implications in the control of ticks and in the epidemiology of tick-borne diseases, particularly in areas where dogs live in close contact with their wild counterparts.

In conclusion, all topics stressed above are worthy of research in the future. Data from these studies would provide new insights into the biology and ecology of *Rh. sanguineus *and ultimately prompt the development of optimized strategies for the control of this tick and the pathogens it transmits.

## Competing interests

The author declares that they have no competing interests.

## Supplementary Material

Additional file 1 **Ecdysis**. This video shows a female during the ecdysis process. Note the contractions of the idiosome, which is driven by regular peristaltic waves, and the exuvia that is being slipped off backwards. Click here for file

Additional file 2 **Moulting of nymphs**. This video display several nymphs during the moulting process. Note a newly moulted nymph literally entering into the exuvia of another nymph, somehow stimulating its emergence. Click here for file

Additional file 3 **Excretion**. This video shows the exact moment of the excretion of a white spherule (faeces) by a female prior to its emergence of the nymphal exuvia. Click here for file
